# Thickening of the greater auricular nerve in leprosy: clinical correlation by ultrasound

**DOI:** 10.1590/0100-3984.2017.0041

**Published:** 2018

**Authors:** Eduardo Kaiser U. N. Fonseca, Felipe Melo Nogueira, Sarah Simaan dos Santos, Tatiana Goberstein Lerner, Adham do Amaral e Castro

**Affiliations:** 1 Escola Paulista de Medicina da Universidade Federal de São Paulo (EPM-Unifesp), São Paulo, SP, Brazil.

*Dear Editor,*

**Figure 1:**
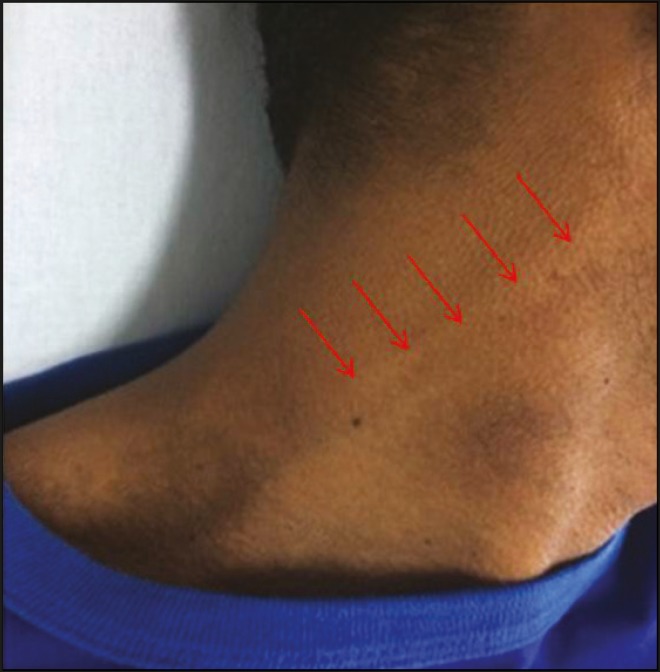
Enlarged (thickened) right greater auricular nerve (arrows).

**Figure 2:**
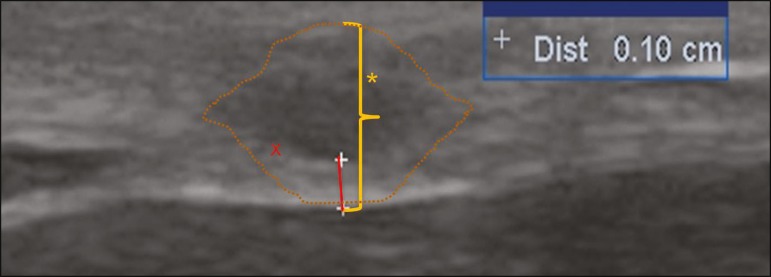
Ultrasound of the right greater auricular nerve (asterisk, with contours indicated by dotted lines), in an axial view, showing a hyperechoic rim, corresponding to the epineurium, with a thickness of 1.0 mm (red x). The cross-sectional area of the nerve was estimated to be 50 mm^2^.

A 39-year-old man presented with a two-month history of painful erythematous-violet nodular lesions on his trunk and lower limbs, accompanied by arthralgia in the small joints of the hands, wrists, ankles, and elbows, with morning stiffness for about an hour that improved with movement. He reported having experiencing similar, self-limited episodes for the last year and a half, each episode lasting for approximately five days and accompanied by significant weight loss. On physical examination, the patient was malnourished, with lamellar desquamation and mobile nodules of approximately 3 cm, together with erythematous-violet lesions on the anterior surface of thighs, just above the knees bilaterally and at the left thoracoabdominal junction. He also had gross thickening of the fibular and ulnar nerves bilaterally, as well as of the right greater auricular nerve (Figure 1). The diagnosis of lepromatous leprosy was confirmed by skin biopsy. We assessed the peripheral nerves by ultrasound (Figure 2).

**Figure 3:**
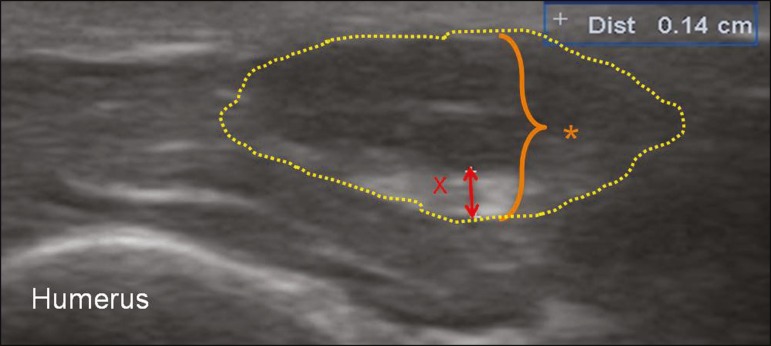
Ultrasound of the left ulnar nerve (asterisk, with contours indicated by dotted lines), in an axial view, 20 mm above the elbow, showing thickening of the epineurium (red ×), which had a thickness of 1.4 mm—much greater than the average (0.77 mm) and similar to the greatest thickness reported by Visser et al.^(5)^.

Ultrasound imaging of the peripheral nerves can be used in order to assess their morphology, identify thickening, estimate the thickness of the epineurium, and calculate their cross-sectional area, as well as to determine their echogenicity and (on Doppler ultrasound) vascularity^(1-3)^. The ulnar nerve is most often affected, followed by the median and fibular nerves^(4)^. However, to our knowledge, there have been no ultrasound studies assessing alterations in the greater auricular nerve, which is involved in 18% of cases^(4)^. In the case presented here, we were able to assess that nerve and found it to be enlarged, as shown in Figure 2. We also identified enlargement of the left ulnar nerve, at approximately 20 mm above the elbow (Figure 3), as was previously reported by Visser et al.^(5)^.

Despite the scarcity of data in the literature on specific quantification of thickening of the greater auricular nerve, we believe that this case illustrates the value of ultrasound in assessing the nerve. Although it is generally smaller than the ulnar nerve, the thickening parameters of the greater auricular nerve epineurium in our patient were similar to the cut-off points for the ulnar epineurium established in other studies^(5)^.

Leprosy is an endemic mycobacteriosis that has a broad clinical spectrum, characterized by nerve and cutaneous lesions with nerve thickening^(1,5)^, and is relatively common in Brazil. Several recent studies have proposed measuring nerve thickness with high-resolution ultrasound involving the use of high-frequency linear probes. That technique has provided a good evaluation of peripheral nerves^(6)^. An increase in the cross-sectional area of the nerve can thus be identified, providing an assessment of the degree of nerve damage, and the technique could be used in follow-up evaluations^(2)^. Here, we have reported the first case in which ultrasound evaluation of the greater auricular nerve revealed its thickening in a patient with leprosy.

The use of ultrasound for determining nerve thickness could significantly improve early diagnosis of peripheral neuropathy in leprosy, because it can show the changes that occur even before nerve thickening is palpable or visible on clinical examination. A major goal of treatment is to prevent nerve damage, which progresses to cause physical disabilities^(1,4)^. In this context, the monitoring of leprosy patients through the use of bedside ultrasound evaluation is a quite useful tool.
